# Prophylactic vs. Therapeutic Effect of Probiotics on the Inflammation Mediated by the NF-κB Pathway in Inflammatory Bowel Conditions

**DOI:** 10.3390/biomedicines11061675

**Published:** 2023-06-09

**Authors:** Saeideh Najafi, Fattah Sotoodehnejadnematalahi, Mohammad Mehdi Amiri, Mohammad Reza Pourshafie, Mahdi Rohani

**Affiliations:** 1 Department of Biology, Science and Research Branch, Islamic Azad University, Tehran 14778-93855, Iran; saeideh.njf@gmail.com (S.N.); sotoudehnejad@srbiau.ac.ir (F.S.); 2 Department of Immunology, School of Public Health, Tehran University of Medical Sciences, Tehran 14155-6619, Iran; amiri.mm@gmail.com; 3 Department of Bacteriology, Pasteur Institute of Iran, Tehran 13169-43551, Iran

**Keywords:** probiotics, inflammatory bowel disease, inflammation, *Lactobacillus*, *Bifidobacterium*, NF-κB

## Abstract

Probiotic supplements consumed adequately at the proper time can affect health by modulating inflammatory pathways in gastrointestinal epithelial cells and modifying the resultant inflammatory response. The current study applied in vitro models to investigate the effectiveness of probiotics in modulating inflammatory pathways and altering inflammatory gene expression in gastrointestinal epithelial cells, with the ultimate goal of promoting probiotic consumption as a therapeutic and preventive measure for chronic inflammatory bowel conditions. HT-29 cells were treated with Gram-negative bacteria to evaluate the changes in pathways related to inflammation activities before and after treatment with a *Lactobacillus* spp. cocktail (*L. plantarum*, *L. rhamnosus*, *L. brevis*, and *L. ruteri*) and a *Bifidobacterium* spp. cocktail (*B. bifidum*, *B. langum*, and *B. breve*) using the real-time PCR method and ELISA for IL-1β and IL-6 as pro-inflammatory cytokines. The results showed that the expression of NF-κB signaling pathway genes and IL-1β and IL-6 cytokines increased after exposure to Gram-negative components. In contrast, all probiotic combinations significantly decreased the expression of genes and the secretion of cytokines. However, this decrease was significantly smaller in cells that underwent probiotic treatment after inflammation induction. In addition, cocktails containing combined *Lactobacillus* and *Bifidobacterium* demonstrated robust anti-inflammatory activity relative to solo cocktails. Our observations confirm that probiotic consumption could positively impact inflammatory conditions and alleviate inflammatory symptoms; they can be particularly effective as a preventive measure. Our study provides preliminary evidence to support the lifetime consumption of probiotics.

## 1. Introduction

Inflammatory bowel diseases (IBD), such as ulcerative colitis (UC) and Crohn’s disease (CD) are chronic inflammatory disorders of the gastrointestinal tract [1]. The incidence and prevalence of IBD have risen across various parts of the world over time, suggesting that IBD is now a global ailment [2]. Although the exact cause of IBD is still unclear and it is believed to have multiple factors, current research indicates that an imbalance between the gut microbiota and the immune response of the epithelial cells is a contributing factor in the development of this disorder [3]. Among the range of treatment options available, probiotics have shown great promise as a safe and effective approach to controlling IBD symptoms [4]. According to the World Health Organization, probiotics are live microorganisms colonizing the gut whose adequate consumption confers health benefits to the host [5]. Most probiotics belong to the *Lactobacillus* and *Bifidobacterium* genera [6]. They have been utilized to modulate inflammation in inflammatory bowel conditions in the last two decades [7,8]. These bacteria function through the interactions of bacterial cell components such as pathogen-associated molecular patterns (PAMPs) with pattern recognition receptors [9] and shift the microflora to beneficial bacteria by preventing pathogenic bacteria’s growth and maintaining the balance of normal intestinal flora [10,11].

There are various studies in the literature discussing the potential benefits of probiotics in relation to IBD. However, there is still a lack of consensus regarding their precise effectiveness. The concept of using probiotics as a treatment and preventive measure for IBD is gaining popularity. VSL#3, a probiotic containing a mix of four strains of *Lactobacillus*, three strains of *Bifidobacteria*, and one strain of *Streptococcus thermophilus*, is the most commonly used, with a confirmed efficacy in IBD [12,13,14,15]. Its use in UC cases has been demonstrated to decrease levels of TNF-α and IL-6 [16]. There has been noticeable symptomatic relief, such as less frequent rectal bleeding. However, since these patients were concurrently taking anti-inflammatory drugs, the positive outcomes could be credited to both the probiotics and the medications [12,13]. The utility of VSL#3 in CD cases has not shown the same efficacy in preventing relapses as it has in UC. Instead, supplementation with *Faecalibacterium prausnitzii* appears to be more successful [17]. IBD is a complex disease with many contributing factors, and the likelihood of identifying a single strain that benefits all patients with the same condition is low [18].

Toll-like receptors (TLRs) and NOD-like receptors (NLRs) are two main families of pattern recognition receptors that detect a wide range of microbial patterns [19]. TLRs transmit signals through the sequential action of myeloid differentiation factor 88 (*MyD88*), tumor necrosis factor receptor-associated factor 6 (*TRAF6*), interleukin receptor-associated kinase 1 and 4 (*IRAK1*, *IRAK4*), and mitogen-activated protein kinase 1 (*TAK1*) [20]. Eventually, *TAK1* activates nuclear factor-kappa B (*NF-κB*) through the phosphorylation and degradation of I-kappa-B (*IκB*) inside the *NF-κB-IκB* complex. In turn, *NF-κB* initiates the transcription of inflammatory cytokines in the nucleus, including tumor necrosis factor-alpha (TNF-α) and various interleukins (IL) such as IL-6 and IL-1β [21,22]. Among the TLRs involved in the NF-κB pathway, *TLR4* and *TLR5* play a vital role in various inflammatory bowel diseases by inducing an innate immune response against Gram-negative lipopolysaccharide (LPS) and flagellin, respectively [23,24,25]. Nucleotide-binding oligomerization domain-containing protein 2 (*NOD2*), which also activates the NF-κB pathway, is a type of NLR that detects intracellular patterns and is activated by a peptidoglycan particle called muramyl dipeptide in inflammatory conditions [26].

Although probiotics have been suggested to control inflammation in bowel diseases [27], no established guidelines on the prophylactic or therapeutic use of probiotics have been published, partly because of the scarcity of studies comparing their efficacy in primary prevention with efficacy in symptom control during active disease. Moreover, since the immunomodulatory effects of each probiotic are strain-specific, exploring novel strains is helpful in uncovering probiotics’ supplementary role in improving inflammatory disease symptoms. To tackle these challenges, the present study employs in vitro models to evaluate the preventive and therapeutic effects of different probiotic combinations that incorporate novel native *Lactobacillus* and *Bifidobacterium* species. To provide a clear image of the effect of probiotics on inflammation, we evaluated the expression of genes in various stages of the NF-κB pathway, including receptors and downstream signaling molecules and the secretion of pro-inflammatory cytokines as final products. The effectiveness of different combinations of *Lactobacillus* and *Bifidobacterium* probiotics was also compared. The effect of probiotics on the modulation of inflammation was also evaluated over time.

## 2. Materials and Methods

### 2.1. Bacterial Strains

In previous studies, we demonstrated the probiotic properties of our native strains of *Lactobacillus* and *Bifidobacterium* isolated from the stool samples of healthy individuals, healthy mothers’ milk, and their healthy infants’ stool [28,29,30]. *Lactobacillus* spp. were isolated from stool samples obtained from 53 Iranian volunteers aged 1 to 36 who did not suffer from any known gastrointestinal disease and had not received any antibiotics in the prior six months [30]. *Bifidobacterium* spp. were isolated from 28 mothers’ breast milk samples and their infants’ feces samples. Mothers and infants did not have any disease up to three months before sampling. Additionally, infants were born through vaginal delivery after a normal full-term pregnancy [29]. Eventually, ten bacterial strains were isolated, including the *Lactiplantibacillus plantarum* PR 365 strain, *Lactiplantibacillus plantarum* PR 42 strain, *Lacticaseibacillus rhamnosus* PR 195 strain, *Levilactobacillus brevis* PR 205 strain, *Limosilactobacillus reuteri* PR 100 strain, *Bifidobacterium bifidum* PR 1063 strain, *Bifidobacterium bifidum* PR 1044 strain, *Bifidobacterium breve* PR 1005 strain, *Bifidobacterium breve* PR 1015 strain, and *Bifidobacterium langum* PR 1001 strain. The plates were incubated in anaerobic conditions at 37 °C for 24 h. 

We also obtained *Salmonella enterica* subsp. *enterica* ATCC 9270 and enterotoxigenic *Escherichia coli* (ETEC) k88 from the Pasteur Institute of Iran Microbial Bank as viable Gram-negative pathogens to induce inflammation in the intestinal epithelial cells.

### 2.2. Cell Line

The HT-29 cell line, derived from human colon adenocarcinoma (NCBI–C466), was purchased from the Pasteur Institute of Iran Cell Bank. The cells were enriched in RPMI-1640 medium (Thermo-Gibco, Grand Island, NY, USA) and 10% bovine fetal serum (FBS) (Biochrom, Berlin, Germany). We also added streptomycin and penicillin antibiotics (Sigma-Aldrich, Gillingham, UK) to prevent infection. The cells were maintained in an incubator with 5% carbon dioxide (CO_2_) at 37 °C and 95% humidity. The culture medium was replaced every other day to achieve 70–90% confluency. 

### 2.3. Preparation of Bacterial Cocktails

To prepare the Gram-negative bacterial components, overnight Luria–Bertani broth (LB broth) cultures (Thermo Fisher Scientific, Waltham, MA, USA) of *Salmonella enterica* and ETEC were adjusted to 0.5 McFarland standard and underwent five cycles of one minute of sonication and one minute of rest. The sonicated mixtures were stored at −80 °C for treatments. 

To prepare the probiotic cocktails, all probiotic strains were incubated in De Man, Rogosa, and Sharpe broth (MRS) (Merck, Darmstadt, Germany) under anaerobic conditions for 24 h at 37 °C. After incubation, the strains were centrifugalized at 8000 rpm for 5 min. The pellets were diluted with antibiotic-free RPMI-1640 medium with 10% FBS, and the optical density was adjusted using an OD_600nm_ spectrophotometer based on the results of the MTT assay as described below. Three probiotic cocktails were prepared: five strains of *Lactobacillus* (homogeneous cocktail), five strains of *Bifidobacterium* (homogeneous cocktail), and a combination of all ten *Bifidobacterium* and *Lactobacillus* strains (heterogeneous cocktail). 

### 2.4. MTT Assay for MOI Determination

The viability of HT-29 cells after exposure to bacteria was assessed using an MTT assay (Bioidea, Tehran, Iran) according to the manufacturer’s instructions. First, a concentration of about 5 × 10^3^/well of HT-29 cells was seeded into a 96-well microplate. Then, different concentrations of bacteria (1 × 10^5^, 1 × 10^6^, 1 × 10^7^, and 1 × 10^8^ CFU/mL of live probiotics) were added to the wells, and the plates were incubated at 5% CO_2_ for 24 and 48 h at 37 °C. Wells containing cells alone were considered as a control. Following incubation, viable cells were evaluated based on absorption readings at 570 nm using an ELISA microplate reader (eBioscience, Inc., San Diego, CA, USA). The results were reported as a biological percentage and IC50 (concentration that inhibits cell growth up to 50%). The MTT experiment was replicated three times, and the cell survival rate was calculated according to the following formula [31,32]:

$$Cell\;survival\;rate=\frac{\left(Sample\;absorbance-Blank\;absorbance\right)}{\left(Control\;absorbance-Blank\;absorbance\right)}\times 100$$

Following the MTT assay, the ratio of 1 × 10^7^ CFU/mL (MOI 10) was determined to be the concentration that inhibits the proliferation of 50% of HT-29 cells after both 24 and 48 h. Therefore, it was used as the desired dose for probiotics.

### 2.5. Challenge with Probiotics and Gram-Negative Bacteria

To model both the primary prevention and treatment of inflammatory bowel conditions, we subjected HT-29 cells to probiotic and Gram-negative component treatments in two setups: pre- and post-treatments, as shown in Table 1, which took place in two stages. In stage 1 of the pre-treatment setup, probiotics were added to each well containing HT-29 cells and incubated for an hour under a humidified atmospheric condition at 37 °C in 5% CO_2_. After incubation, treated cells were washed twice with warmed phosphate-buffered saline (PBS) at pH 7.4. Then, antibiotic-free RPMI with a 10% FBS medium was added to each well. In stage 2, Gram-negative bacteria components were added and incubated for an additional five hours. An opposite sequence of steps was performed for the post-treatment setup. After incubation, wells were washed with PBS four times to detach unattached bacteria. Then, antibiotic-free RPMI with 10% FBS medium was added to each well and incubated at 37 °C in 5% CO_2_ for 24 and 48 h.

Some cells were also treated solely with Gram-negative bacteria as a positive control, and cells without further treatment were considered a negative control. The supernatants of the incubated cells were collected at 24 and 48 h and centrifuged at 8000 rpm for 5 to 6 min. The supernatants were recovered and stored at −20 °C for ELISA. The treated cells were also detached using 0.25% Trypsin-EDTA (Gibco, Grand Island, NY, USA) and centrifuged at 8000× *g*. The pellet was harvested for RNA extraction. The immunomodulatory effect of the probiotic cocktails was evaluated at 24 and 48 h.

### 2.6. Total RNA Isolation and cDNA Synthesis

According to the manufacturer’s instructions, total RNA was isolated from treated HT-29 cells (approximately 106 cells) using a High Pure RNA Isolation Kit (Roche Co., Mannheim, Germany). A Nanodrop 1000 UV Vis Spectrophotometer measured the absorbance of purified RNA to determine the RNA’s purity. Additionally, electrophoresis on 2% agarose gel was performed to examine the integrity of the RNA. Finally, cDNA templates were synthesized from the whole RNA using a cDNA synthesis kit (Yekta Tajhiz Azma, Tehran, Iran) according to the manufacturer’s protocol. We stored cDNA samples at −20 °C.

### 2.7. Quantitative Real-Time PCR (qRT-PCR)

We examined the expression of the *TLR4*, *TLR5*, *NOD2*, *MyD88*, *IRAK1*, *TRAF6*, *TAK1*, *NF-κB*, *IL-1β*, and *IL-6* genes through quantitative real-time PCR. In addition, glyceraldehyde-3-phosphate dehydrogenase (GAPDH) was used as a housekeeping gene. The qPCR primer sequences were retrieved from the online Primer Bank website (http://pga.mgh.harvard.edu/primerbank, accessed on 7 August 2020), as shown in Table 2. The material required for each qRT-PCR reaction with a final volume of 20 µL was as follows: 10 µL of 2× real-time PCR master mix (SYBR Green) (Takara Bio, Kusatsu, Japan), 1 μL of forward primer (10 pm/μL), 1 μL of reverse primer (10 pm/μL), 2 µL of cDNA, and 6 µL sterile distilled water. The thermal cycler Stratagene Mx3000p (Santa Clara, CA, USA) was set at 95 °C for 10 min to initiate the polymerization process, followed by 40 cycles of amplification at 95 °C for 15 s, proper annealing temperature (Table 2) for each primer for 30 s, and 72 °C for 30 s. Relative mRNA expression was calculated using the formula RQ = 2^−ΔΔCt^ [26].

### 2.8. Cytokine Assays

A phenotypic survey to measure the production of pro-inflammatory cytokines was performed using an ELISA kit (Karmanian Pars Gene, Rafsanjan, Iran). According to the manufacturer’s protocols, the supernatants of probiotics and pathogen-treated HT-29 cells were collected to measure IL-6 and IL-1β levels.

### 2.9. Statistical Analysis

Data analysis was performed using SPSS software version 16 (SPSS Inc., Chicago, IL, USA). The statistical significance of differences was determined using Student’s *t*-test and one-way ANOVA followed by the Tukey HSD post hoc comparison test. A level of significance (*p*-value) less than 0.05 was considered significant. All results are reported as mean ± SD.

## 3. Results

**Figure 1:**
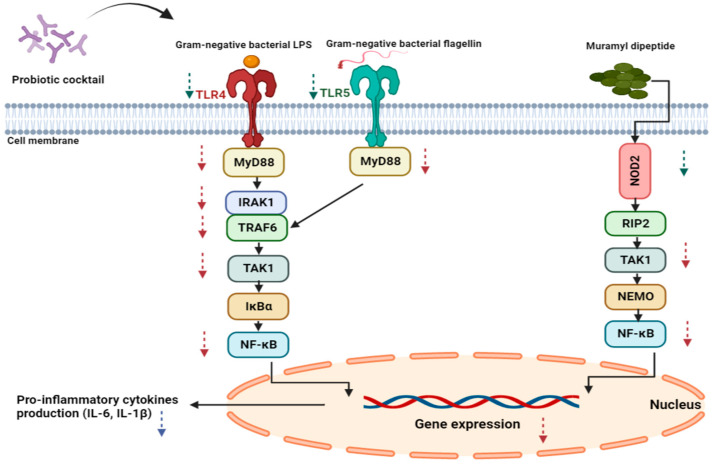
The addition of probiotics inhibits the effect of Gram-negative bacterial PAMPs in both setups by downregulating the expression of *TLR 4* and 5, *NOD2*, and downstream signaling molecules, with a final reduction in pro-inflammatory cytokine production. The green arrows show changes in receptor gene expression, while the red arrows indicate changes in the expression of downstream molecules’ genes. Lastly, the purple arrow represents a change in cytokine production.

In the current study, we compared the effect of different combinations of native *Lactobacillus* and *Bifidobacterium* species on the NF-κB pathway, including the expressions of *TLR4*, *TLR5*, and *NOD2*, downstream cell signaling genes *MyD88*, *IRAK1*, *TRAF6*, *TAK1*, and NF-κB, and the level of IL-1, and IL-6 pro-inflammatory cytokines’ secretion. The effect of probiotics on the NF-κB signaling pathway is illustrated in Figure 1. The probiotic efficacy was compared in different exposure patterns to Gram-negative bacteria to assess changes in inflammation over time.

### 3.1. Receptors and NF-κB Pathway Modulation

**Figure 2:**
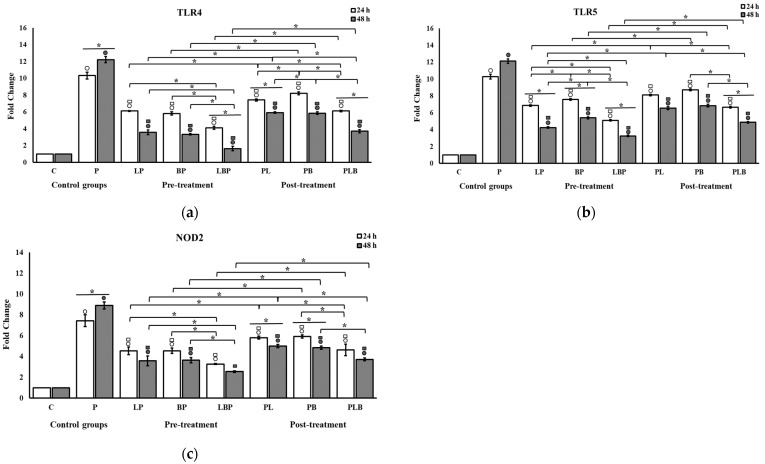
The effect of *Lactobacillus* and *Bifidobacterium* cocktails on the mRNA expression of (**a**) *TLR4*, (**b**) *TLR5*, and (**c**) *NOD2* in HT-29 cells exposed to Gram-negative bacteria. Gene expression levels were measured using qRT-PCR after 24 and 48 h of treatment. All results are reported as mean ± SD (n = 3) and *p* < 0.05. C: control; P: pathogen; L: *Lactobacillus*; B: *Bifidobacterium*; the order of letters indicate treatment order (e.g., BP indicates pre-treatment cocktail of *Bifidobacterium*). ○ indicates significant difference to control (C). □ indicates significant difference to pathogen (P). * indicates significant difference between treatment setups.

The effects of probiotics on the mRNA expression of *TLR 4* and 5 and *NOD2* receptors are shown in Figure 2. HT-29 cells challenged with Gram-negative bacteria responded by significantly increasing the expression of *TLR4*, *TLR5*, and *NOD2* receptors in both time intervals, especially after 48 h. The addition of probiotics significantly dampened this response consistently in all cocktails, though it was more prominent in the heterogeneous cocktails (*p* < 0.05). The significance of differences between the *Lactobacillus* and *Bifidobacterium* homogeneous cocktails was not consistently observed in all setups. The post-treatment setup was consistently less effective than the pre-treatment setup in reducing receptor gene expression (*p* < 0.05). On the other hand, the expressions were inclined to reduce more on the second day, and the differences from the first day reached significant levels (*p* < 0.05) in half of the treatments.

**Figure 3:**
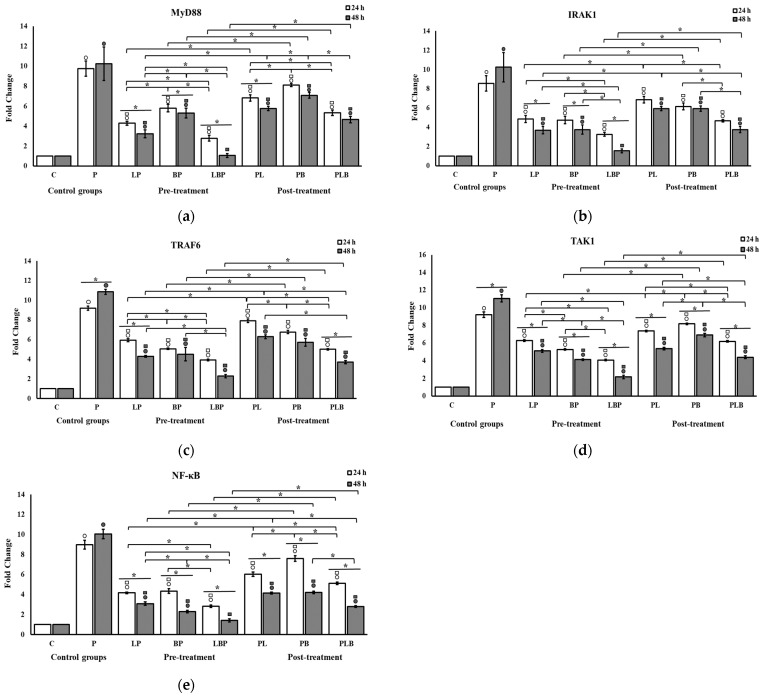
The effect of probiotic cocktails on the expression level of downstream genes related to the NF-κB pathway (**a**) *MyD88*, (**b**) *TIRAF6*, (**c**) *IRAK1*, (**d**) *TAK1*, and (**e**) *NF-κB* in HT-29 cells infected with Gram-negative bacteria. Gene expression levels were measured using qRT-PCR after 24 and 48 h of treatment. All results are reported as mean ± SD (n = 3) and *p* < 0.05. C: control; P: pathogen; L: *Lactobacillus*; B: *Bifidobacterium*; the order of letters indicate treatment order (e.g., BP indicates pre-treatment cocktail of *Bifidobacterium*). ○ indicates significant difference to control (C). □ indicates significant difference to pathogen (P). * indicates significant difference between treatment setups.

The effects of the probiotics on the expression of downstream genes in the NF-κB pathway are shown in Figure 3. Gram-negative bacteria significantly stimulated the expression of all downstream genes, including *MyD88*, *IRAK1*, *TRAF6*, *TAK1*, and *NF-κB* in the NF-κB pathway, in both time intervals, especially 48 h after treatment in all setups. In contrast, probiotics significantly inhibited their expression in all cocktails (*p* < 0.05). The probiotic inhibition was significantly more effective in heterogenous cocktails relative to homogenous cocktails (*p* < 0.05) except in the post-treatment setup of the MyD88 gene at 48 h, in which the difference between the heterogenous cocktail and the *Bifidobacterium* homogenous cocktail was not significant (Figure 3a). Interestingly, the heterogenous probiotic cocktail was able to decrease the expression to levels at which no significant difference was observed relative to control cells in the pre-treatment setups of the *MyD88*, *IRAK1*, and *NF-κB* genes after 48 h (*p* > 0.05) (Figure 3a,b,e). The significance of differences between the *Lactobacillus* and *Bifidobacterium* homogeneous cocktails was not consistent across the setups. The post-treatment setups demonstrated a significantly smaller decrease in gene expression compared to the pre-treatment setups (*p* < 0.05). The level of mRNA expression reduction was more pronounced on the second day. However, these differences from the first day were not significant in 9 of the 30 treatments.

### 3.2. Pro-Inflammatory Cytokine Modulation by Probiotic Cocktails

**Figure 4:**
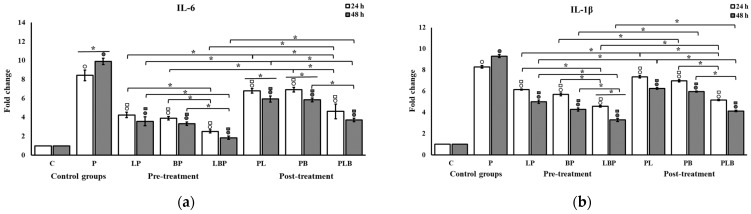
The effect of probiotic cocktails on the expression of (**a**) *IL-6* and (**b**) *IL-1β* cytokines in HT-29 cells infected with Gram-negative bacteria. Gene expression levels were measured using qRT-PCR after 24 and 48 h of treatment. All results are reported as mean ± SD (n = 3) and *p* < 0.05. C: control; P: pathogen; L: *Lactobacillus*; B: *Bifidobacterium*; the order of letters indicate treatment order (e.g., BP indicates pre-treatment cocktail of *Bifidobacterium*). ○ indicates significant difference to control (C). □ indicates significant difference to pathogen (P). * indicates significant difference between treatment setups.

The effects of the probiotics on the mRNA expression level of pro-inflammatory cytokines are shown in Figure 4. Cells challenged with Gram-negative bacteria without probiotic treatment consistently demonstrated a significant increase in the expression of *IL-6* and *IL-1β* mRNA compared to the control cells at both 24 and 48 h (*p* < 0.05). Moreover, probiotic treatments significantly reduced the level of cytokine gene expression in all cocktails compared to the cells exclusively challenged with the Gram-negative component (*p* < 0.05). Significantly more reduction was seen when cells were treated with the heterogeneous cocktails compared with the homogeneous cocktails at both time intervals (*p* < 0.05). The decrease in the expression of cytokines under the influence of either the *Bifidobacterium* or *Lactobacillus* homogeneous cocktails showed no significant difference at any time after treatment (*p* > 0.05). The post-treatment setups demonstrated a significantly smaller decrease than the pre-treatment setups at both 24 and 48 h (*p* < 0.05). The reduction in cytokine expression was observed on the second day with more intensity than on the first day, though the difference was not significant in most of the treatments.

**Figure 5:**
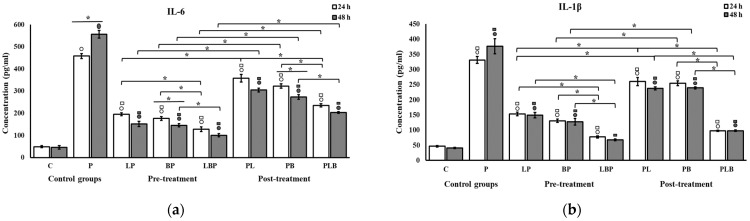
The effect of probiotic cocktails on the secretion of (**a**) IL-6 and (**b**) IL-1β cytokines from HT-29 cells exposed to Gram-negative bacteria. Cytokine levels were measured using ELISA after 24 and 48 h of treatment. All results are reported as mean ± SD (n = 3) and *p* < 0.05. C: control; P: pathogen; L: *Lactobacillus*; B: *Bifidobacterium*; the order of letters indicate treatment orders (e.g., BP indicates pre-treatment cocktail of *Bifidobacterium*). ○ indicates significant difference to control (C). □ indicates significant difference to pathogen (P). * indicates significant difference between treatment setups.

The effects of the probiotics on the production of pro-inflammatory cytokines are shown in Figure 5. The production of cytokines was significantly higher after the challenge with the Gram-negative bacteria (*p* < 0.05). Conversely, probiotics significantly decreased cytokine production in the HT-29 cells in all treatments (*p* < 0.05). Unlike the results of RT-PCR, the significantly higher effectiveness of the heterogenous cocktail in terms of the reduction in cytokine production relative to the homogenous cocktail was not consistently observed across the setups. However, after the addition of the heterogenous cocktail in the pre-treatment setup, IL-1β production reached levels at which no significant difference from control cells was observed after 48 h (*p* > 0.05) (Figure 5b). The homogenous cocktails in the post-treatment setup were less effective at reducing cytokine production relative to the pre-treatment setup (*p* < 0.05), but this significance was not invariably present for the heterogenous cocktails in IL-1β (*p* > 0.05). The differences between production levels on the first and second day were not significant (*p* > 0.05), except for the IL-6 secretion of the homogenous *Bifidobacterium* cocktail in the post- and pre-treatment setups (Figure 5a).

## 4. Discussion

The majority of inflammatory conditions of the intestinal tract, such as chronic inflammatory bowel diseases, are characterized by an imbalance between pro-inflammatory and anti-inflammatory cytokines, leading to a hyper-inflammatory state [33]. In chronic inflammatory bowel diseases such as CD and UC, the substitution of normal intestinal flora with pathogens, particularly Gram-negative bacteria, results in dysbiosis [34]. The activation of TLR4 and TLR5 by the PAMPs of these pathogens changes the immunological balance toward a hyper-inflammatory state [35]. Moreover, a strong correlation was seen between CD and the expression of NOD2 receptors. The activation of TLRs and NOD2 synergistically induces the NF-κB pathway. The induction of the NF-κB pathway results in the upregulation of inflammatory cytokines [36,37]. Among these cytokines, the essential role of IL-6 and IL-1β in uncontrolled inflammation is well known in chronic inflammatory bowel diseases [38]. Therefore, treatment strategies targeting these molecules have been used to control inflammation in these conditions [39]. It is speculated that some probiotics can counteract the NF-κB pathway to maintain immune hemostasis [40]. To compare the effectiveness of our novel probiotic strains on the primary prevention of inflammation vs. inflammation control in already inflamed cells, we used pre-treatment setups as an in vitro model for prevention and post-treatment setups as an in vitro model for inflammation control.

In our study, HT-29 cells demonstrated an increased pro-inflammatory response when exposed exclusively to sonicated *Salmonella enterica* and the ETEC k88 strain. The gene expression of *TLR4*, *TLR5*, *NOD2* receptors, and the associated downstream intracellular genes, *MYD88*, *IRAK1*, *TRAF6*, *TAK1*, and *NF-κB*, along with the final pro-inflammatory products IL-1β and IL-6 upregulated in the presence of Gram-negative bacteria. The amplification of gene expression led to a rise in the secretion of IL-1β and IL-6 cytokines. In contrast, we demonstrated that exposure to probiotics abated the inflammatory response to the Gram-negative bacterial component. The gene expression of the aforementioned receptors, proteins, and cytokines was downregulated, and the secretion of final cytokines dropped.

Through engagement with TLRs and NLRs, probiotics interact with intestinal epithelial cells, consequently modulating downstream cell signaling molecules involved in NF-κB pathways [41]. In our HT-29 cells, the interaction of probiotics with these receptors led to a decreased expression of downstream molecules. Eventually, the inhibition of NF-κB signaling leads to decreased secretion of pro-inflammatory cytokines and the attenuation of inflammation. It is noteworthy that studies on some probiotics did not elicit a similar anti-inflammatory response, which indicates that these effects are highly dependent on the genus, species, and strain of probiotic bacteria [42]. For instance, Chapman et al. investigated 15 probiotics and observed significant variations among probiotic genera and species in terms of their abilities to inhibit inflammation caused by various pathogens, including *E. coli*, *S. typhimurium*, and *C. difficile* [43]. Some investigators suggested that the differences in biological effects among probiotics may be related to the secretion of factors such as bacteriocin, lactic acid, and short-chain fatty acids [44]. This heterogeneity among probiotics emphasizes the significance of studying the effect of native probiotics on gastrointestinal epithelial cells to discover new probiotic profiles for the management of inflammatory bowel diseases.

Furthermore, we observed that the pre- and post-treatment setups followed the same trend of reducing inflammation. However, the gene expression in post-treatment setups was reduced less than in pre-treatment setups, suggesting that probiotics’ efficacy decreases for inflammation control in already inflamed cells, and they are more helpful when used for primary inflammation prevention. Similarly, Duary et al. explored the effect of *L. Plantarum* (Lp9, Lp91, and Lp5276) in LPS-stimulated HT-29 cells in pre- and post-treatment setups, and their results showed that gene expression was significantly reduced by the *Lactobacillus* strains in the pre-treatment setups. Interestingly, the researchers observed an opposite effect in the post-treatment setups [45]. Although their results corroborate our findings that probiotics are more effective for primary prevention than active disease treatment, the increase observed in post-treatment setups in their study, unlike our study, provides further evidence that probiotic effects are strain-specific.

In the current study, we compared the heterogenous cocktails of *Bifidobacterium* and *Lactobacillus* probiotics with homogenous cocktails to evaluate the effect of these probiotics on each other. Our data demonstrated a stronger effect on inhibiting the expression of pro-inflammatory genes when combining *Lactobacillus* and *Bifidobacterium* for the majority of our genes. Interestingly, *NOD2*, *MyD88*, *IRAK1*, and *NF-κB* gene expression reached the levels of control cells in pre-treatment setups after 48 h, suggesting our heterogenous cocktails’ superiority in modulating the immune response at equal OD. Since each probiotic species may involve different mechanisms to impact epithelial cells that can potentiate each other’s effect, the immunomodulatory response may be more pronounced when several probiotics are taken together and act synergistically [46]. Nevertheless, after 48 h of the cocktails’ surveillance, the absence of significant cytokine secretion changes relative to the homogenous cocktails did not corroborate this finding. Studies comparing the impact of single- and multi-strain probiotics in gastrointestinal inflammatory disease are limited and have shown conflicting results. Our result appears to be consistent with the findings of Li et al., who compared the effect of heterogenous cocktails of *L. acidophilus* and *B. animalis* with homogenous cocktails in infected HT-29 cells and similarly concluded that heterogenous cocktails are more potent in decreasing *NF-κB*, *MAPK*, and *IL-8*, while no difference was seen between *L. acidophilus* and *B. animalis* homogenous cocktails [47]. Chapman et al. similarly showed that a mixture of probiotics is more effective than each probiotic alone at reducing inflammation. However, their conclusions also differed from ours as they inferred that single-strain *Lactobacillus* cocktails are more effective than *Bifidobacterium* [43]. Other studies conducted by Candela et al. and Sheikhi et al. were not in agreement with our findings as they did not observe any synergistic effect for heterogenous cocktails [48,49].

In this study, the anti-inflammatory response to probiotics showed a propensity to be enhanced over time. A meta-analysis of the effect of the duration of probiotic treatment for gastrointestinal disease showed that the treatment efficacy increases over time. After several weeks of treatment, this advantageous time effect was significant [50]. In the current study, the reduction in gene expression was more noticeable on the second day. Although the difference from the first day was not significant in many setups, the downward trend may have become significant if the gene expression had been evaluated for a more extended period.

Our study has certain limitations. Firstly, we were unable to ascertain the levels of protein production associated with the anti-inflammatory properties of these strains, a task typically undertaken through methods such as blotting. In addition, we did not utilize in vivo methods to substantiate the anti-inflammatory effects of these strains from a molecular standpoint. Nevertheless, despite these limitations, the in vitro clarification of distinct molecular mechanisms could potentially yield valuable insights into the unique properties of these probiotic strains.

## 5. Conclusions

This study contributes valuable insights into the role of probiotics in managing inflammatory bowel conditions. Our findings suggest a superior effectiveness of our *Lactobacillus* and *Bifidobacterium* probiotics in primary prevention over their efficacy during active disease treatment. This suggests a potential direction for future preventative strategies against inflammatory bowel conditions. Our unique combination of *Lactobacillus* and *Bifidobacterium* was found to exert an enhanced anti-inflammatory effect in inflamed epithelial cells, marking a significant contribution to the understanding of probiotic combinations in inflammation control. These findings expand our knowledge of the mechanism of action of probiotics by demonstrating a comprehensive reduction in the expression of pro-inflammatory genes and secretion of pro-inflammatory cytokines in inflamed HT-29 cells. Significantly, we found that the anti-inflammatory benefits of our probiotics increase with the duration of treatment, adding to the limited body of evidence on the temporal effects of probiotics on inflammation. Therefore, this study opens up the potential for a new understanding of long-term probiotic use and its potential role in improving overall inflammatory conditions. Our research underscores the therapeutic potential of specific probiotic combinations in preventing and managing inflammatory bowel conditions. Further studies, however, are needed to validate these promising findings and their clinical implications.

## Figures and Tables

**Table 1:** Treatment setups of HT-29 cells.

Treatment Setups	Description
Pre-treatment	LP: HT-29 cells challenged with Gram-negative bacteria after Lactobacillus cocktail treatment
	BP: HT-29 cells challenged with Gram-negative bacteria after Bifidobacterium cocktail treatment
	LBP: HT-29 cells challenged with Gram-negative bacteria after Lactobacillus and Bifidobacterium cocktail treatment
Post-treatment	PL: HT-29 cells treated with Lactobacillus cocktail after Gram-negative bacteria challenge
	PB: HT-29 cells treated with Bifidobacterium cocktail after Gram-negative bacteria challenge
	PLB: HT-29 cells treated with Lactobacillus and Bifidobacterium cocktail after Gram-negative bacteria challenge

**Table 2:** Primer sequences of reference and target genes used for qRT-PCR.

Name	Forward Primer (5′ > 3′)	Reverse Primer (5′ > 3′)	Product Size (bp)	Tm (°C)	Primer Bank ID
*GAPDH*	GGAGCGAGATCCCTCCAAAAT	GGCTGTTGTCATACTTCTCATGG	197	61	378404907c1
*TLR4*	AGACCTGTCCCTGAACCCTAT	CGATGGACTTCTAAACCAGCCA	147	61	373432602c1
*TLR5*	TCCCTGAACTCACGAGTCTTT	TGGTTGTCAAGTCCGTAAAATGC	109	61	281427130c3
*NOD2*	TGGTTGGTTCAGCCTCTCACGATGA	CAGGACACTCTCGAAGCCTT	157	61	11545911c1
*MyD88*	GGCGGCTGCTCTCAACATGCGA	CTGTCTGTGTCCGCACGTTCAAGA	61	61	289546652c1
*IRAK1*	TGAGGAACACGGTGTATGCTG	GTTT GTTTGGGTGACGAAACCTGGA	119	61	68800242c2
*TRAF6*	TTTGCTCTTATGGATTGTCCCC	CATTGATGCAGCACAGTTGTC	120	61	332000008c2
*NF-κB*	GAAGCACGAATGACAGAGGC	GCTTGGCGGATTAGCTCTTTT	137	56	259155300c2
*TAK1*	ATTGTAGAGCTTCGGCAGTTATC	CTGTAAACACCAACTCATTGC	186	66	21735563c2
*IL-1β*	ATGATGGCTTATTACAGTGGCAA	GTCGGAGATTCGTAGCTGGA	132	61	27894305c1
*IL-6*	ACTCACCTCTTCAGAACGAATTG	CCATCTTTGGAAGGTTCAGGTTG	149	61	224831235c1

## Data Availability

The data that support the findings of this study are available from the corresponding author upon reasonable request.
